# Multiple Congenital Epulis in Alveolar Ridges of Maxilla and Mandible in a Newborn: A Rare Case Report

**DOI:** 10.1155/2014/606985

**Published:** 2014-04-08

**Authors:** Nader Saki, Somayeh Araghi

**Affiliations:** ^1^Department of Otolaryngology, Head and Neck Surgery, Hearing & Speech Research Center, Ahvaz Jundishapur University of Medical Sciences, P.O. Box 61537-15794, Ahvaz, Iran; ^2^Department of Otolaryngology, Head and Neck Surgery, Hearing & Speech Research Center, Imam Khomeini Hospital, Ahvaz Jundishapur University of Medical Sciences, P.O. Box 61537-15794, Ahvaz, Iran

## Abstract

Congenital granular cell lesion (CGCL) or congenital epulis is an uncommon benign of the oral cavity tumor appearing at birth with typical clinical and pathologic features. It predominately affects females, mainly on the anterior maxillary alveolar ridge, and occurs usually as a single mass, although sometimes as multiple. The left side incisor area is the most common site. The etiology and histogenesis of the lesion remain obscure. Though it is a benign lesion, the tumor may cause feeding and respiratory problems if there are too large or multiple tumors. We report a case of a three-day-old, female newborn, who was referred to our hospital with multiple congenital oral swellings arising from the maxilla and mandible. The tumors caused a feeding problem and, hence, they were resected by surgical excision under general anesthesia.

## 1. Introduction

Congenital epulis (CE) is an uncommon benign tumor. This condition clinically appears as a protuberant mass in a round or ovoid shape, pedunculated, or sessile [1]. The CE normally arises from the anterior part of the maxillary alveolar ridge of the newborn and frequently laterals to the midline in the area of the developing primary lateral incisor and canine [1, 2]. Other than gingiva, a few cases of congenital epulis have been reported on the tongue [3, 4]. Congenital granular cell epulis (CGCE) typically presents as a tan-pink polypoid mass with a smooth nonulcerated surface. The CGCE is composed of large, polygonal granular cells, characterized by an abundance of eosinophilic granular cytoplasm and round or oval basophilic nuclei. These distinctive cells grow in a sheet-like pattern supported by delicate fibrovascular septa. Incorporation of odontogenic epithelium is occasionally seen. The overlying surface epithelium is usually intact and atrophic. The cut surface is homogeneous, firm, and tan to yellow [5]. In this case report we report a rare case of CE, a newborn with multiple CE in both maxillary and mandibular alveolar ridge that interfered with feeding.

## 2. Case Report

**Figure 1:**
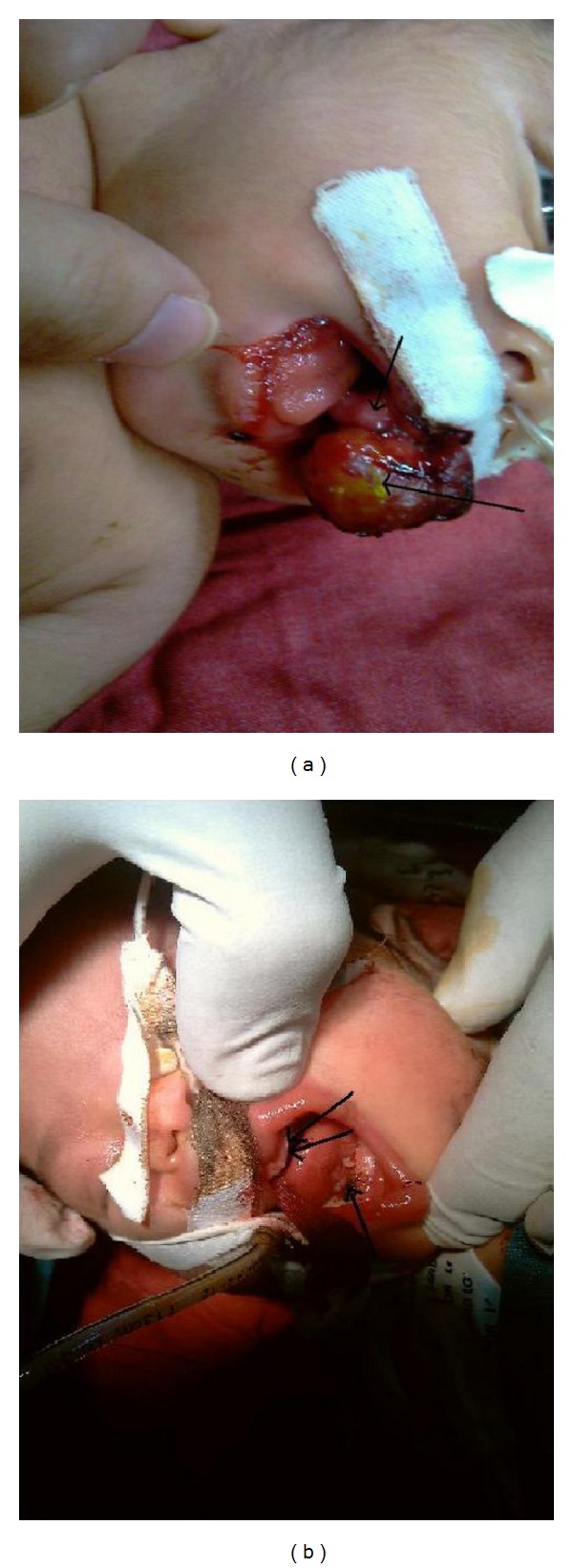
A large pedunculated mass and two other smaller tumors on the maxillary and mandibular alveolar ridges in a neonate female (before and after resection).

A female newborn was referred from a pediatric hospital for the treatment of soft tissue masses on the alveolar ridges of the maxillary and mandibular noticed at birth (Figure 1). She was a three-day-old infant, term, from a cesarean section delivery with 3200 gr birth weight, 48 cm birth height, and 34 cm head circumference. Her mother was a 30-year-old G2P1Ab0 woman. All prenatal evaluations such as sonographic findings were normal and no abnormality was reported. The family history of congenital disorders was negative and three soft tissue masses on the alveolar ridges were seen. The biggest one (2∗1.5∗1 cm) was protruding from her mouth and arising from the mucosa of the upper alveolar ridge of the left side of the maxilla. It was interfering with the feeding but did not cause airway obstruction. Another mass was located medial to the biggest one which was explained above (1∗0.5∗0.4 cm) and the third one (1∗0.8∗0.5 cm) was located on the lower alveolar ridge of the left side of the mandible. Otherwise, the infant was healthy. The patient underwent the surgical excision of the three masses (Figure 1). The postoperative period was uneventful.

**Figure 2:**
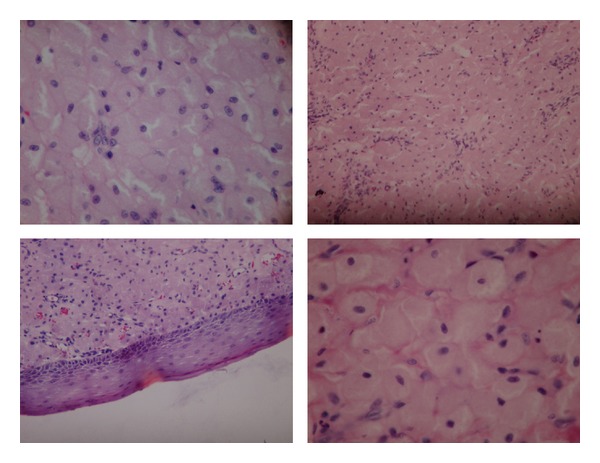
Histopathologic slides.

Grossly, in consistency, the masses were oval, creamy and firm and the external surface was slightly irregular. Microscopically, sections revealed neoplastic cells with round small nuclei and abundant granular cytoplasm. Among the tumoral cells, thin vascular plexus was seen. The external surface was covered by stratified squamous epithelium (Figure 2). This classical histological pattern is indicative of congenital epulis.

## 3. Discussion

This benign tumor which appears rarely has various names such as congenital epulis (CE), congenital gingival granular cell tumor of the newborn [3], or Neumann's tumor [6].

In a study, in 2005, Olson, Marcus, and Zuker reported that CE is seen three times more frequently on the anterior alveolar ridge of the maxilla than from the mandible with a female to male ratio of 8 : 1 with a Caucasian predisposition [7]. In another study Kannan and Rajesh said that the condition is not associated with any other dental abnormalities or congenital malformations. Usually, it appears as a single lesion, but in 10% cases it may arise from multiple locations simultaneously [8]. The size of the tumor may vary from a few millimeters to 9 cm. Due to the ineffective deglutition and postnatal feeding, breathing difficulties, and also interference with mouth closure, larger lesions may cause prenatal hydramnios [8]. Our case presentation of a CE was a female Persian neonate diagnosed with three pedunculated masses, two in the anterior maxillary alveolar ridge and one on the anterior mandibular alveolar ridge and all were located at the left side. The lesions were interfering with her feeding.

Prenatal ultrasound evaluations have been used to report congenital epulis in sporadic cases after the 25th week of gestation, but the findings are nonspecific, and ultrasonic differential diagnoses, including congenital malformations and various benign and malignant intraoral tumors; however, it can help in the management of pregnancy, delivery, and postnatal management especially if the lesion is large [8]. In our case, the prenatal ultrasound evaluation did not reveal any intraoral mass and polyhydramnios.

Treatment consists of CE removal by surgery under local or general anesthesia [1, 2]. In a report lesions were surgically excised between two days and six weeks from birth (median, 5.5 days) [4]. The treatment adopted in this case was the surgical excision in the fourth day under general anesthesia because the lesions were interfering with feeding. Hiradfar M et al. reported a 15-day-old female infant with multiple congenital epulis of the mandibular alveolar ridge in 2012.

It is important to stress that clinicians should know differential diagnoses of growths in the oral cavities of newborns, including hemangioma, lymphangioma, fibroma, granuloma, rhabdomyosarcoma, and osteogenic and chondrogenic sarcomas; schwannoma and heterotopic gastrointestinal cyst as the treatment modalities will be different for each case [1, 2]. In the present case, the clinical diagnosis of CE was further confirmed by the histopathological examination of the fibrotic mass removed from the patient, which showed a stratified squamous mucosa and a prominent branching fibrovascular network.

Although benign, it may obstruct the oral passage by its size and induce breathing and feeding problems. Delay in operation may cause airway obstruction and feeding difficulty. The tumor should be removed during the immediate postnatal period, as in our case.
